# Users’ experience of community-based power assisted exercise: a transition from NHS to third sector services

**DOI:** 10.1080/17482631.2021.1949899

**Published:** 2021-07-12

**Authors:** Rachel Young, David Broom, Rachel O’Brien, Karen Sage, Christine Smith

**Affiliations:** a Department of Allied Health Professions, Faculty of Health and Wellbeing, Sheffield Hallam University, Sheffield, UK; b Academy of Sport and Physical Activity, Faculty of Health and Life Sciences, Coventry University, Coventry, UK; c Department of Nursing, Faculty of Health, Psychology and Social Care, Manchester Metropolitan University, Manchester, UK

**Keywords:** Stroke, community-based venue, assisted exercise, qualitative, interview, phenomenology

## Abstract

**Purpose:**

Seated Power Assisted Exercise (PAE) equipment is an accessible exercise mode for people with limited mobility following stroke and is available at a small number of community-based venues. The purpose of this qualitative study was to understand the lived experience of using PAE amongst PwS in a community venue and identify recommendations for the development and advancement of PAE equipment.

**Method:**

Semi-structured interviews were conducted with 8 participants (PwS) attending a community stroke venue where PAE equipment was available. Transcribed data were analysed using interpretative phenomenological analysis.

**Results:**

Three overarching themes emerged from the analysis; 1) *Don’t tell me I’ve plateaued*; 2) *PAE facilitates the transition into long-term recovery*; 3) *Reframing the experience of stroke*. Participants associated the uptake of PAE alongside venue membership as a turning point in their adjustment to life following stroke. In addition, recommendations for future development of the equipment were identified.

**Conclusion:**

These findings indicate that membership of a stroke venue alongside engagement with PAE facilitated transition from early stroke rehabilitation into longer term recovery. The results of this study have informed the need for future product design and highlighted PAE is an effective mode for continued rehabilitation in third-sector services.

## Introduction

Worldwide, there are over 13 million new cases of stroke annually and it is a leading cause of adult disability (Johnson et al., 2019; Lindsay et al., 2019). Although physical rehabilitation services are typically finite (Miller et al., 2019), People with Stroke (PwS) have long-term potential for neuroplastic adaptation and functional recovery (Bunketorp-Käll et al., 2017; Sun & Zehr, 2019). Strength, aerobic and functional exercise interventions are known to improve mobility, reduce physiological risk profiles and enhance participation for at least five years beyond the onset of stroke (D’Isabella et al., 2017; Poltawski et al., 2015; Saunders et al., 2016; Young et al., 2019). Supporting PwS to transition from rehabilitation services into longer term exercise programmes has been identified as a priority within published guidelines (MacKay-Lyons et al., 2020; Royal College of Physicians, 2016). Purposed services and venues that specifically target the needs of PwS are limited (Schouten et al., 2011) and the need for closer partnerships between health care and community wellness programmes has been identified (Miller et al., 2019).

Qualitative research has facilitated an understanding of and insight into the experiences of exercise amongst PwS and service providers (Condon & Guidon, 2018; Signal et al., 2016); the barriers to engagement in exercise following stroke include beliefs about personal capability and availability of accessible resources (Nicholson et al., 2014). Group sessions in de-medicalized external venues are associated with enhanced life participation and self-efficacy (Poltawski et al., 2015) and a systematic review of the qualitative literature indicated that perceived benefits may outweigh measured physical impact (Young et al., 2019). However, fitness professionals report that they have limited confidence in supporting the specific needs of PwS (Condon & Guidon, 2018) and inaccessible equipment has been identified as a barrier to the uptake of exercise amongst the stroke population (Nicholson et al., 2014). Exercise programmes can be effectively delivered in the home environment (Galvin et al., 2014) but barriers to exercising at home include fear of falling, caregiver addressing other priorities, unsuitable environment and limited confidence with correct exercise technique (Galvin et al., 2014; Scorrano et al., 2018).

People with severe or complex physical impairment following stroke are under-represented in exercise research due to the challenges associated with access and physical ability (Saunders et al., 2016). The ability to walk independently has been a fundamental inclusion criterion for participation in most exercise trials for PwS (Galloway et al., 2019; Marzolini et al., 2020) and thereby excludes those individuals with limited mobility. The challenges associated with promoting and enabling exercise amongst non-ambulant PwS are particularly complex (Valkenborghs et al., 2019). Advances in assistive interventions and adapted models of service delivery are essential to enable people with moderate or severe motor impairment to experience physical training following stroke (Kerr et al., 2019; Stoller et al., 2013).

Improvements in physical function and exercise behaviours are reported amongst people with complex impairments who have participated in adapted or assisted exercise interventions (Kerr et al., 2019; Lloyd et al., 2018). The impact of assisted pedalling interventions for PwS include increased aerobic capacity, enhanced neuroplasticity and improved motor coordination (Holzapfel et al., 2019; Linder et al., 2019, 2015). Seated Power Assisted Exercise (PAE) machines go beyond assisted pedalling as the range of equipment is designed to assist repeated, multi-directional, global movement patterns. The equipment has been adopted by providers of rehabilitation and therapy services who have identified the potential benefit of multi-directional assistive equipment (West Berkshire Therapy Centre (WBTC), 2016). Investigation into the feasibility of PAE for people with complex neurological impairment recorded a 100% programme completion rate, 96% attendance at sessions in which the participants engaged with and complied with all prescribed exercises and no serious adverse events were recorded (Young et al., 2018). Participants in the trial reported perceived improvements in their physical and psychosocial wellbeing. An enhanced understanding of the user experience is required to optimize design and implementation of the equipment for complex populations.

To date, research on assisted exercise following stroke has focussed on quantified physical impact of laboratory-based-assisted pedalling and there is a paucity of qualitative research on the perceived effects of assisted exercise or the experience of accessing community-based venues. The motivators for and perceived value of self-initiated engagement with PAE following stroke have not been elucidated. Participation in exercise is an individualized experience influenced by numerous intrinsic and extrinsic factors including physical ability, self-efficacy and environment (Eynon et al., 2019). Qualitative exploration of the lived experience can facilitate a comprehensive understanding of the individual, their context and the influence of the setting; which in turn, may inform the effective development, implementation and evaluation of rehabilitation programmes (Merali et al., 2019).

Interpretative phenomenological analysis (IPA) is a qualitative approach which explores the reported lived experience and is well suited to the exploration of health behaviours and novel concepts (Smith, 2007). By capturing the essence of the lived experience, IPA has previously facilitated the development of stroke rehabilitation strategies which enhanced user empowerment and optimized achievement of meaningful outcomes (Garrett et al., 2011; Williams & Murray, 2013). IPA emphasizes an individualized approach towards data analysis to gain an understanding of the factors influencing their reported experience (Alase, 2017) and has been previously applied to the development of exercise interventions amongst populations with complex needs (Wheeler et al., 2018). Using IPA, this article addresses the gap in the qualitative literature on assisted exercise at a community-based venue by reporting on the lived experiences of people who use PAE equipment; exploring perceived physical and psychosocial outcomes associated with this form of exercise amongst ambulant and non-ambulant PwS.

## Aims

The aim of this study is to explore both ambulant and non-ambulant PwS experiences of and the perceived effects associated with participation in PAE in a third sector community stroke centre. After interpretation, user-centred recommendations for the development and advancement of PAE equipment will be compiled.

## Methods

This study received ethical approval from Sheffield Hallam University (registration number ER6774925). The data management plan ensured compliance with General Data Protection Regulations (GDPR).

### Methodological approach

Given the complexity and uniqueness of the experiences and perceived effect of using PAE equipment amongst people with stroke, IPA was used to facilitate an appreciation of individuals’ values and meanings (Alase, 2017). This study was grounded within the interpretivist paradigm, focussing upon contextual factors and the mutual interdependence between causes and effects. The ontological assumptions which underpinned the study were relativist, recognizing that the reality of human experience is subjective; the epistemological position was founded upon the construction of knowledge through human interaction between the researcher and participants (Cuthbertson et al., 2019). IPA is well suited to understanding health behaviours and recognizes interconnectedness between the individual and the world (Smith, 2018a). Stroke is a complex condition, affecting multiple components of physical and psychosocial wellbeing; outcomes should not be considered in isolation but in the context of the whole person and their wider circumstances (Pringle et al., 2013).

Application of the hermeneutic cycle in IPA ensures that the parts are related to the whole, and the whole to the parts, enabling a holistic and context centred understanding of human experience (Smith, 2007). Interpretative studies typically recruit a small, homogenous sample to facilitate an in-depth and rich understanding of the phenomena of interest in the context of participants’ broader life experiences (Smith et al., 2009). IPA is based on the premise that people continually reflect upon and make sense of their experiences; the role of the interviewer in IPA is to invite the participant to share their view of the world and how they make sense of it (Smith, 2018a).

### Sample population, venue and recruitment

Convenience sampling in qualitative research facilitates the identification of people with specific life experience relevant to the research aim (Newton-John, 2018). It was first necessary to identify an operator which offered PAE equipment for use by PwS. The machines manufactured by Shapemaster Global LTD represent a unique design in the global marketplace due to the combination of multi-directional assisted movements which enable the user to simultaneously engage all four limbs and the trunk whilst seated. Through consultation with the manufacturer, the research team identified a third sector (registered charity independent of government) venue in the north of England dedicated to the long-term support of PwS. A visit to the centre was arranged during which the lead author met with the team and took the opportunity to view the gymnasium area which comprised six seated PAE pieces of equipment, a motorized treadmill, assisted pedalling machine (Moto-Med) and static parallel bars. The centre offered a range of membership packages and support services including physiotherapy; PwS in the locality could self-refer or be signposted by a healthcare professional. Membership criteria were wholly inclusive to people with any type or severity of physical, communication or cognitive impairments following stroke, although attendees were required to resource their selected package. This may have been a barrier for attendance for people from economically disadvantaged households.

A poster and flyers were placed in the venue and the site manager circulated participant information sheets to interested members. Variation in age and stroke severity and a combination of male and female participants were sought to capture diverse experiences. Meetings were scheduled between potential participants and the lead author (RY) by the centre manager to enable the opportunity for members to explore any questions and understand the requirements of participation. The meetings facilitated the development of a relationship between the participants and the interviewer to build trust and enhance the richness of data shared during the subsequent interview (Morse, 2015). This informal meeting enabled the interviewer to screen eligibility for inclusion in the study and, on completion of written, informed consent, a date and time for each interview based at the centre was scheduled.

### Inclusion and exclusion criteria

The inclusion criteria were as follows: a diagnosis of stroke confirmed by the centre manager; experience of using PAE within the previous 12 months; ability to provide informed consent and ability to communicate verbally in spoken English at a level comprehensible for an audio recording. Specific frequency or duration of PAE engagement was not stipulated as this was explored during data collection. The exclusion criterion was an inability to respond to verbal questions for the purposes of an interview.

### Power assisted exercise

**Figure 1.**
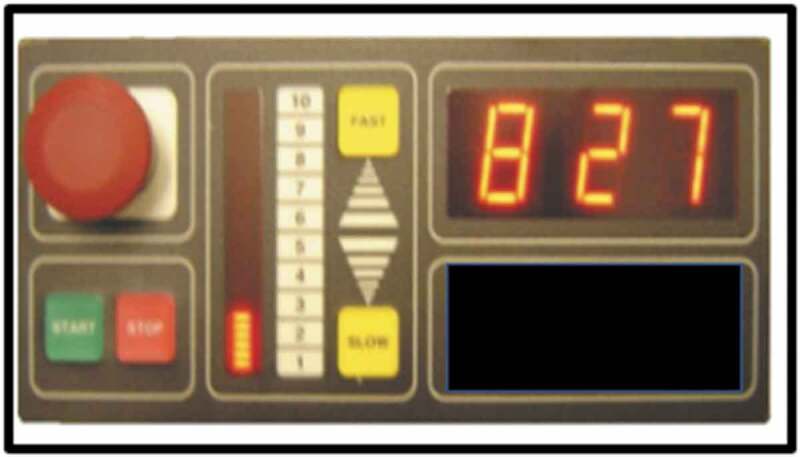
Console of the PAE machine

Six seated PAE machines were sited at the venue, the combined limb and trunk movements assisted by each machine are described in Table I. The machines were operated by a console which featured start/stop and speed settings between one and ten (Figure 1). The default duration of each machine was five minutes; a workout using all six machines would typically take 35–40 minutes including time to transfer on and off the equipment. In order to use the equipment safely, members needed to have independent sitting balance and weigh less than 24 stone (152 kg). Non-ambulant people could use a transfer aid to access the equipment. Users were supervised by support staff at the venue and encouraged to generate a physical effort by moving with the machine.

**Table I.** PAE machines

Machine Name	Actions assisted	Image
**Chest and Legs**	Shoulder; mid-range flexion and extension Elbow; mid-range flexion and extension Hip; mid-range flexion and extension Knee; flexion and extension	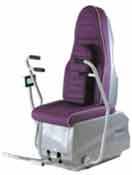
**Seated climber**	Shoulder; flexion into elevation Elbow; flexion into end range extension Hip; mid-range flexion and extension Knee; flexion and extension	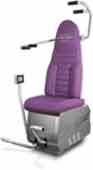
**Seated abductor**	Hip; abduction and adduction Shoulder; horizontal abduction and adduction	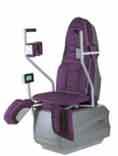
**Side bend stepper**	Trunk; lateral flexion Reach or push down through arms Hip; flexion and extension Knee; flexion and extension	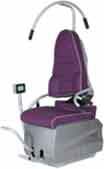
**Rotatory torso**	Trunk rotation Elbows and shoulders supported in flexion	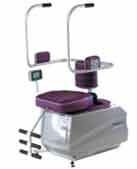
**Tummy crunch**	Trunk; flexion and extension Hips are flexed Shoulders are elevated	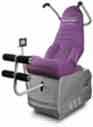

### Data collection

Baseline demographic data were recorded by the first author and included participant’s age, gender, ethnicity, time since stroke and occupation. Participants were allocated a numerical code to ensure anonymity.

PAE equipment at the venue was used by people who were independently mobile alongside people with very limited mobility. A baseline measurement of mobility was recorded using the Functional Ambulation Category (FAC) (Mehrholz et al., 2007). The FAC is an ordinal scale which allocates mobility on a spectrum of 0 to 5; a score of 0 indicates that the individual cannot walk, whilst a score of 5 is allocated to people who can walk independently anywhere. The FAC was used to capture an overview of functional ambulation and explore whether severity of impairment influenced the reported lived experience of using the equipment. Observation of walking ability was scored by the lead author according to the FAC on recruitment to the study. The lead author observed each participant performed a transfer between seated surfaces and attempted to walk along a flat 5-m walkway in the centre. A member of the centre team who had moving and handling skills and knowledge of the individual participant supervised or assisted the assessment as required. Two participants were able to perform these tasks independently and were subsequently observed whilst attempting to walk up and downstairs to determine whether they gained a score of 4 or 5.

Semi-structured interviews were conducted by the lead author who is a neurological physiotherapist with 25 years’ experience working in clinical practice and higher education with a specific interest in exercise interventions for PwS. The interviews were scheduled at a mutually convenient time and based in a quiet space within the venue. Carers or partners were invited to accompany the interview and their comments were included in the analysis. Recent debate has criticized IPA as a “therapy-oriented” research methodology rather than a phenomenological approach (Van Manen, 2017). The data captured during our study were for the purpose of understanding the use of PAE amongst PwS to address a current gap in the qualitative literature. The lead author was mindful of her background as a therapist (albeit physical rather than psychological) and used a reflective journal to promote a sustained focus on data collection rather than counsel during the interviews.

An interview schedule containing open questions was developed by the research team and used in a flexible manner; questions explored pre-stroke lifestyle, memories of early stroke recovery and experiences of engaging with PAE to capture an understanding of how uptake of PAE related to the wider experience of living with stroke (appendix 1). The interviews were audio-recorded on an Olympus digital voice recorder (WS-811) and brief field notes on observed, non-verbal communication were documented by the interviewer. Thereafter, all interviews were transcribed verbatim by the lead author using Microsoft Word software to facilitate emersion and familiarization with each item.

The value of member checking in ensuring rigour in qualitative research has been questioned (Smith & McGannon, 2018). However, given the communication and cognitive changes that can occur following stroke, participants were given the opportunity to comment on their transcript and check for accuracy. The transcripts were returned to each participant between two and four weeks following the interview. No amendments were requested.

### Data analysis

IPA aims to provide an account of participant experiences and is underpinned by a process of coding, organizing, integrating and interpreting data (Smith et al., 2009). Three members of the research team (RY, KS, RB) adopted an idiographic approach towards data analysis which comprised repeated reading of individual transcripts to familiarize with the participants and gain a context-oriented understanding of their account. Content which explored recall of early stroke, the initiation of PAE and how this influenced the reported experience of stroke recovery was organized through development of preliminary descriptive codes. Subsequent linguistic coding identified subtleties in the data, for example, how participants applied different types of deictic reference during the interview which facilitated an interpretative insight into their respective sense of empowerment at different stages of stroke recovery. The descriptive and linguistic codes were expanded to create conceptual codes which captured an impression of the reported lived experience and enabled the research team to identify individual emergent themes. Connections between emergent themes were grouped into units of meaning alongside supporting quotations (Smith et al., 2009). The ideographic approach was sustained through the development of an interpretative summary for each participant which encapsulated their experience of PAE embedded within the context of their pre-stroke self, early rehabilitation, current mobility, social and cultural influences. While cross-case analysis in IPA has not aimed to generate broad generalizations, a theoretical transferability of findings might be expected to emerge (Smith et al., 2009). Cross-case analysis allowed for recognition of shared and divergent experiences across the sample and tendencies associated with age, gender, occupation, time since stroke and mobility across the data set were explored and emergent trends were tabulated. Multiple superordinate themes which reflected the recall of stroke onset, hospital, rehabilitation and uptake of PAE were distilled to create overarching themes rooted in the context of each individual account and the shared but varied experiences of assisted exercise following stroke.

### Rigour

Data collection and analysis were based on a relativist position with an emphasis upon fidelity to the insights and experiences shared by the participants and utility in terms of the synergic relationship between the research design and aims (Smith & McGannon, 2018). A responsive approach was adopted during each interview to enhance credibility of the data generated and this ideographic approach was sustained throughout the data analysis. The lead author adopted a reflexive approach through continuously evaluating potential biases which may have influenced the interpretation of the data and recording key insights in a reflective journal (Berger, 2013). Confirmability of the research was enhanced through regular peer debriefing with the research team and analytical triangulation (Morse, 2015). Two co-authors (KS, RO) independently coded their allocated transcripts before discussing their individual interpretation with the lead author. An equal voice was given to all three members of the coding team to promote rigour in triangulation (Smith & McGannon, 2018). Strong conflict in opinion did not arise but the three authors shared alternative emphases in perspective which enriched the interpretative analysis. The final overarching themes represented the integrated interpretation of all three members of the coding team.

## Results

Nine members of the third-sector centre expressed interest in participation after viewing the poster. All met the inclusion criteria and provided their informed consent; one participant dropped out prior to interview due to illness that was not related to participating in PAE.

The study recruited eight PwS (6 M; 2 F) who were regular members at the third-sector centre. The mean age in years was 60.1 years (range 42–76, SD 10.69) and mean time in months since stroke was 48 (range 14–90 months SD 31.92). At the time of having their first stroke, four participants were in employment, three participants were retired and one participant was two-week postpartum (Table II). One participant had experienced a second stroke. Two male participants (M2, M5) attended the interview with their female partner. Mean interview duration in minutes was 35.59 (range 18.23–55.26, SD 10.79). All participants stated their ethnicity as white British. Seven participants presented with hemiparesis and one participant was ataxic (Table III). Functional ambulation category (FAC) scores ranged from 0/5 to 5/5 (mean 2.87).

**Table II.** Summary of demographic and condition-related data

	Mean (SD)
**Age (years)**	60.12 (10.69)
**Time since stroke (months)**	48.12 (31.92)
	**Number (%)**
Gender Male Female	**6 (75%)** **2 (25%)**
Marital status Married/partnership Separated/divorced Single	**4 (50%)** **2 (25%)** **2 (25%)**
Employment status Employed (paid) Unable to work (disability) Retired	**0** **5 (62.5%)** **3 (37.5%)**
Ethnicity White British	**8 (100%)**
Functional Ambulation Category 0–2 3-4 5	**3 (37.5%)** **3 (37.5%)** **2 (25%)**

**Table III.** Individual condition and impairment data

Code	Age	Gender	Time since stroke (months)	Stroke impairment	FAC
M1	53	Male	16	Right hemiparesis	3/5
M2	76	Male	42	Left hemiparesis	0/5
M3	68	Male	48	Right hemiparesis	3/5
M4	52	Male	82	Ataxia	3/5
M5	62	Male	14	Left hemiparesis	2/5
M6	62	Male	18	Left hemiparesis	2/5
F1	42	Female	98	Right hemiparesis	5/5
F2	66	Female	67	Right hemiparesis	5/5

All participants had been attending the venue for at least six months; frequency of visits ranged from two to five sessions per week. Seven out of the eight participants reported using the equipment during each visit, the eighth participant was adjusting to a bereavement at the time of interview and had not used the machines for the two months prior to interview. The recall of pre-stroke lifestyle and account of current routines were summarized for each participant alongside a pseudonym (Table IV) to facilitate an ideographic visualization of the data throughout the results section.

**Table IV.** Expanded individual data and context

Participant	Lifestyle prior to stroke	Current lifestyle	Centre membership	Therapy and exercise routine
**M1** **(Mike)**	Employed as a senior manager in industry. Was in the process of moving house when stroke occurred. Recalled being very sporty as a young male but had “no time spare” during adult life.	Lives with wife who goes out to work and was described as the “breadwinner.” Grown up children live further afield.	Signposted at the end of NHS rehabilitation by Occupational Therapist approximately one year prior to interview. Visits the centre 2–3 days per week.	X2 sessions of PAE per visit (4–6 per week). 10 minutes on assisted pedals per visit. Unable to use treadmill. Physio session x1 per month.
**M2** **(Tom)**	Retired joiner and craftsman. Active retirement including holidays abroad, DIY and gardening.	Lives with wife and has carers to help with ADL twice per day. Goes to church and enjoys frequent visits from family and friends.	Daughter had heard about it through an incidental conversation whilst out shopping. Joined the centre one year prior to the interview. Visits the centre twice per week.	X1 session of PAE per visit (2 per week). 5 minutes on assisted pedals per visit. Unable to use treadmill. Intermittent access to NHS physiotherapy.
**M3** **(Syd)**	Retired IT consultant and analytics manager. Had been retired for four years prior to stroke. Active retirement including gardening, DIY, lip reading course and busy social life.	Lives with wife and is independent in all self-care. Active member of PPI and service user groups in the area. Visits the centre twice per week and also does Pilates.	Was initially introduced to the equipment at Sheffield Hallam University and an incidental conversation with a taxi driver made him aware of the centre and its facilities.	X2 sessions of PAE per week. 2 × 10 minutes on assisted pedals per week. Unable to use treadmill. Attends pilates x1 per week at a separate venue.
**M4** **(Pete)**	Self-employed as a tradesman. Busy family life with two teenage children. Loved sport, especially golf.	Unable to work as tradesman since stroke. Volunteers at a charity shop twice a week and enjoys seeing his now adult children.	Heard about it through a family friend. Visits the centre five days per week.	X5 sessions of PAE per week. Arrives early to access machines and sometimes extends to 10 minutes per machine. Does not use assisted pedals. Unable to use treadmill.
**M5** **(Colin)**	Retired falconer. Frequent holidays to USA with his wife to visit son. Busy social life with friends.	Gradual resumption of social contact with friends. Feels unable to travel overseas.	NHS psychologist recommended the centre. Visits the centre twice per week.	X2 sessions of PAE per week. 2 × 10 minutes on assisted pedals per week. Unable to use treadmill.
**M6** **(George)**	Employed as an administrator for electronics company. Enjoyed visiting art galleries and often walked lengthy distances between exhibitions. Lived with elderly parents.	Unable to return to work. Tried to resume catching bus to the shops but fearful of falling and also experiences narcolepsy on occasions. Lives with elderly mum as father passed away. Self -caring in ADL.	Stroke Association representative recommended the centre. Joined approximately one year prior to interview. Currently visits twice per week.	X2 sessions of PAE per week. 2 × 10 minutes on assisted pedals per week. X2 sessions with the centre physio per month.
**F1** **(Liz)**	Two weeks post-partum when stroke occurred. Active lifestyle which included running a small holding and frequent exercise sessions.	Initial impairment was severe, has gradually regained mobility and now walks 10 km per day. Still lives at the small holding and is mother to her son and a volunteer at the centre.	Ex-partner had heard about the centre and she joined approximately two years following the stroke. Attends as user and volunteer helper 4–5 days per week.	X4 sessions PAE per week. Does not use assisted pedals or treadmill.

### Key findings

All participants reported perceived value in the combined package of exercise facilities in a supportive, social venue. Although the PAE equipment was a key incentive for joining the centre, the exercise experiences reported were intrinsically connected with peer relationships and appreciation for the team employed at the centre. Analysis of emergent themes highlighted a collective determination to sustain long-term recovery; uptake of membership at the stroke centre was recalled as a brave and positive step towards reframing the lived experience of stroke. Three overarching themes evolved through cross-case analysis of the data set which encompass multiple experiences associated with stroke recovery and PAE; 1) Don’t tell me I’ve plateaued, 2) PAE facilitates the transition into long-term recovery, 3) Reframing the experience of stroke. Patterns associated with FAC score and gender were identified; previous occupation, age or time since stroke did not appear to influence the reported experience.

### Overarching theme 1: don’t tell me I’ve plateaued

Overarching theme one encapsulated the reported experience of hospital-based care and adjustment to life back at home. There was exploration of the expectations associated with stroke recovery and resistance to the concept of recovery plateau was expressed by several participants.

### Hospital was safe but sedentary

The overriding impression of hospital-based care was of feeling safe but disempowered. Recall of hospital was as a sedentary period with rehabilitation limited in terms of quantity and clinician led. Wendy had experienced two strokes and recalled;

*Very little (rehabilitation) at the hospital, you know, they are limited, **they** give you a bit of a walk up and down. (Wendy, 66)*

The third person reference to hospital-based rehabilitation was echoed by Colin;

*In hospital, **they** did physio with me, and I walked in the bars, I used to walk along with them. But not very much. (Colin, 62)*

The rehabilitation teams were recalled in extrinsic terms; they were perceived as people living outside the participants’ experiences. Syd described how he resisted offers of help from hospital staff in order to pursue his own recovery of independence;

*I spent about two hours one day sitting on the side of my hospital bed, trying to put my socks on. But I wouldn’t let any of the nurses come and help. (Syd, 63).*

Timescales along the care and recovery pathway were recurrent themes mentioned by most participants with specific recall of duration of admission and discharge dates. The stay was likened to a period of captivity from which they were eventually “released.” Mike commented;

*It took forever … … . end of June before I was released (from hospital (Mike, 53)*

The term “released” was also used by another male participant, George (62), in the context of his referral to the early supported discharge team which he termed the “*early release scheme.”*

### Rediscovering self

Discharge from hospital to the home environment was pivotal and symbolized a shift towards ownership over recovery, particularly amongst the male participants. Recovery of mobility was recalled in specific detail amongst those participants with lower FAC scores. The language used suggested that rehabilitation became an intrinsic experience rather than something “done unto” them, reflecting a gradual transition from an external to internal locus of control.

*I started trying to walk (without frame) in the house, it’s easier said than done. (Mike, 53)*

Pete recalled challenges associated with his home environment which he had not anticipated during his hospital stay. He was determined to recover enough mobility to avoid extensive structural adaptations to the property;

*About three weeks in I got up steps and started sleeping upstairs, I got up and down, with help of course, only once a day. (Pete, 52)*

In common with other participants, Mike and Pete recalled their hospital stay in third-person terms. The shift to first-person language signifies a sense of empowerment and meaningful engagement with rehabilitation, exercise and goals within the home environment. Guidance from professional teams was valued particularly by those participants with more severe mobility impairment.

Although transition to the home environment was recalled in positive terms, it was also described as a challenging time. Adjusting to the effects of stroke and rebuilding a new way of life was hard; participants viewed recovery as an indefinite process, recovery timescales were uncertain and there was an expectation that change would continue to occur over a long time period.

NHS rehabilitation services were continued through community and out-patient therapy for all participants. This was highly valued with specific memories of individual therapists and meaningful achievements. However, a sense of abandonment could be detected upon discharge from NHS rehabilitation. Liz, who was also adjusting to early motherhood alongside the stroke recalled;

*Home, then six weeks physio and OT and all manner of things and then that stopped (Liz, 42)*

The six-week rehabilitation package was also reported by Mike;

*You’re left to your own devices … .six weeks training and then that’s it. There’s no more and you’re left to get on with. (Mike, 53)*

### Recovery beyond rehabilitation

The participants felt that there was nowhere else to go and four out of the eight people interviewed had heard about the stroke centre through informal word and mouth rather than being signposted by rehabilitation professionals. Colin recalled his discharge from out-patient therapy with feelings of anger and frustration;

*They dumped me! I had to listen to it … … I was really pissed off because they said “you’re beginning to plateau” … … ah hell. (Colin, 62)*

The suggestion of recovery plateau conjured a sense of despair and anger; his emotions were projected towards the rehabilitation team and invoked a determination to prove that he was able to make further improvements. A sense of resistance was shared by several male participants against the expectations communicated in the earlier stages of recovery; rather than accepting the limitations imposed by stroke, there was a determination to explore their abilities and recover their pre-stroke sense of self;

*I’ve made the necessary steps to move away from hospital and be back in charge again (laughs) … . well kind of (Mike, 53)*

The importance of continual goals was emphasized, particularly amongst those with low FAC scores;

*Never give up, you’ve got to have something, if it’s only a small thing, something to keep achieving for, to keep looking for. (Tom, 76)*

The terms “back in charge” and “keep achieving” indicate a sense of autonomy and empowerment which had been recouped following stroke.

Syd described himself as having an analytical approach to all situations and articulated his perspective that recovery following stroke was stepped rather than linear;

*The truth is that you have a slowing down of your progress, it’s almost as if there’s a re-grouping, and then when you feel internally comfortable then you start improving more. (Syd, 68)*

The suggestion here was that the body and brain needed time to adjust to improvements, stabilize and prepare for further changes. A strong commonality across all eight participants was a firm belief that they were still making progress; a combination of intrinsic drive and external support was reflected in their accounts of stroke and recovery. All participants described active lives prior to experiencing stroke; committed to jobs, sport, family or leisure activities. There were no clear patterns associated with the nature of previous occupation and the perceived experience of using the equipment, but the intrinsic drive recognized in their pre-stroke self was evident in the approach taken towards stroke recovery;

*I was hellbent on sport when I was young, now I’m hellbent on getting better. (Mike, 53).*

In summary, overarching theme 1 highlights that the early recovery pathway following stroke comprised in-patient care in hospital which was recalled as a provider of extrinsic support where participants felt safe but sedentary; followed by community rehabilitation, where a sense of ownership over progress was facilitated by therapy teams. The provision of a time limited service was significant to participants, and one participant, Tom, experienced considerable anger and frustration at the point of discharge from rehabilitation. The concept of recovery plateau evoked strong emotional responses amongst some male participants with the perspective that the term was misused to justify termination of therapy.

### Overarching theme 2: PAE facilitates the transition into long-term recovery

All participants reported experience of PAE on the machines sited at the stroke centre. One participant (Wendy) shared that she was not currently using the equipment due to a recent bereavement; the other seven participants were regular users, with use ranging from two to six exercise sessions per week.

### Accessible, user-friendly equipment

The accessibility of the seated PAE equipment in terms of ease of mounting, combined with the assistive mechanism were features recognized by several participants. Participants reported that less mobile people were able to transfer onto the equipment from wheelchairs or through using stand aid equipment such that the opportunity to exercise was opened up in a way which might not have been feasible in a conventional gym setting. Syd and Pete compared the PAE equipment to the facilities available in a standard gym;

*Somebody who has had a stroke just cannot get on the equipment because there is no adaptations, because, once you sit on the bicycle for instance, there’s nothing to stop you from falling off; there’s no adaptations. (Syd, 68)*

*I used to go to the local leisure centre, er but I would say that I could go on the low bike … .there were a few that you could sit on and do things like that … .but I’ve never really seen the variety like they’ve got here. To me, what they’re got here on the different machines, they haven’t got that at other gyms. (Pete, 52)*

The benefits of assisted movement for people with motor impairment included initiation of movement and the ability to exercise for a longer duration.

*It helped me to find the movement again, my right arm just started to join in more and more. (Wendy, 66)*

Differences between participants were detected when they were probed about their favourite machine. Each participant expressed a preference for a specific machine and no clear pattern or consistency was expressed across the data set. Some value was recognized by most participants for all six machines and Syd applied his analytical skills to explain variability of preference according to his current goals;

*Some of the machines were more beneficial to me because they coincided with the goal that I had in place at the time. They were moving the muscles that I was trying to … . Over time, because the goals have changed, so have my favourites changed, because I know I need to do something, how do I achieve it. (Syd, 68).*

Participant variation was also evident with regard to preferred speed settings on the machines. Some participants felt that the faster settings stimulated a higher intensity workout, whilst others thought that slower speeds encouraged them to work harder. When asked about limitations associated with the equipment, several male participants thought that visual feedback on effort generated would be valued. Quantified data pertaining to physical performance would enable users to track progress over time and incentivize sustained engagement with the equipment.

*I’d like to see the boxes (consoles) changed over from what you see now to show how much input you’ve actually put into the machine, so you can see if you’ve actually put input in or whether the machine is just working itself. (Mike, 53).*

There was some comparison between the PAE equipment and the standard treadmill available at the centre. Most participants were unable to access the treadmill due to the severity of their mobility impairment and, for Colin, the treadmill symbolized a meaningful goal. He had attempted to use it but found it too difficult. His partner commented;

*He got quite obsessed with wanting to try it (the treadmill) but then upset when I couldn’t actually do it. (Colin’s partner)*

### PAE as a facilitator of neuromuscular improvement

The overriding sentiment expressed by the participants was that PAE is a facilitator for continued physical recovery following stroke. The accessible design combined with the assistive mechanism opened up an opportunity to exercise for people with limited mobility and motor impairment. Several participants emphasized the importance of user engagement, a balance between the external assistance of the equipment and intrinsically generated effort.

*It’s you who has to put the effort in … .it’s hard, very, very hard. I’ve noticed here, the individuals who go on the machines, do want to learn, they do want to recuperate. (Mike, 53)*

The interviews probed participants to consider the perceived physical effects of PAE. A range of changes associated with the equipment were reported including improved strength and reduced joint stiffness amongst those participants with lower FAC scores; improved control of movement was emphasized by participants with higher FAC scores. The importance of joining in with the movement generated by the machines was emphasized;

*The machines made me stronger … .using your strength to push them has helped … … you feel breathless. (Mike, 53)*

Perceived increases in strength were expressed by several participants and reflections upon improved movement control were insightful, indicating an understanding of the physiological adaptations which occurred during stroke recovery. The concept of neuroplasticity was applied by some participants;

*You’ve got to re-bond, make new tramlines in your head that allow you to use your legs and arms. (Mike, 53)*

In-depth insights into the effect of the equipment on movement and muscular activation were described. Challenges associated with the ability to inhibit muscle activity following stroke were identified and it was felt that PAE could improve motor control;

*By using the equipment I found I was able to shorten the gap between telling the muscles to switch off and them actually switching off. (Syd, 68)*

PAE was directly associated with upper limb recovery by Wendy following her second stroke. She had initially required assistance to maintain contact between her right hand and the exercise machines; through repetition she experienced meaningful improvement in her ability to use her right arm;

*My right arm, I even found it hard holding a cup of tea and carrying it. So now I can do that … . one of the girls held my hand onto the machine for me, initially and then I could do it myself. And I think my face must have been like those that first walk. (Wendy, 66)*

The multi-directional combination of movements assisted by the six machines was viewed as advantageous. Participants described feeling looser following their exercise sessions with perceived benefit from the sensory stimulus. George specifically focussed on improvements in pain symptoms associated with the equipment, stating;

E*xercise it tends to help, to decrease my sensitivity. It’s not numb, I’m not sure how to describe it, tickly I suppose. (George, 62)*

In summary, overarching theme 2 highlights that PAE was associated with perceived improvements in strength, movement control, flexibility and mobility. The exercise represented a forum for social engagement and the physical changes attained through PAE are associated with positive changes in lifestyle and participation for both male and female participants of all ages and abilities.

### Overarching theme 3: reframing the experience of stroke

Reflection upon the physical stimulus of PAE was interwoven with the wider social experience of membership at the centre. This final overarching theme explores transition into a third sector centre for PwS, illustrating the importance of context and environment when evaluating an exercise intervention.

### PAE as a social facilitator

Several male participants recalled that the equipment was a key incentive for taking up membership at the centre; they had not sought a social network, just somewhere to exercise and progress their physical recovery. However, the peer support generated through use of the equipment was valued by all participants and a key incentive to continued engagement. Comparison of ability and recovery between users was evident, particularly amongst those participants with lower FAC scores;

*There’s some a lot worse than me and some a lot better than me … those that are a bit better give you encouragement you see, and those that are worse than me I hope I give them encouragement as well (Tom, 76)*

*I talk to them and like to see how they’re progressing on the machines. And it’s good to hear them say how good I’m doing, it’s good to hear it. (Colin, 62)*

The company of other users also served as a positive distraction from the exercise, making the minutes go faster and created an element of fun alongside the activity.

PAE was associated with positive changes in participation and lifestyle. Female participants and partners reflected upon resumption of family and social activities. Wendy shared that she had recovered her identity as “fun grandma” and talked about gaining the confidence to use a local green gym with her grandchildren;

*There’s a park and over the other side they’ve put those exercise machines for everyone to use. … .and they used to have me going on those, ‘come on grandma, you do it at your group so do it here,’ it’s great fun to do it outside and with them. So if I’d not been using the gym here I would not have ever attempted those there. (Wendy, 66).*

Family relationships were also emphasized by Liz (42) who stated that her son was at the centre of all of her goals and her drive for recovery and improvement.

Changes in social engagement were also recognized by Colin’s partner; she specifically recalled;

*One of our friends, she owns a pub so since the stroke we go in there periodically and she said, only a couple of weeks ago, “I’ve really seen an improvement in him recently, he’s a lot more engaged in what he’s doing, and he’s sitting up straighter.” (Colin’s partner)*

A recurrent sentiment was enjoyment; participants looked forward to their exercise sessions. This reflected the value of wider social and support services surrounding the equipment. Although the PAE machines were an incentive for membership, people could attend the centre knowing that the option to use the equipment was flexible according to what was appropriate for them at any given time.

### We’re lucky to have this place

All participants expressed that they felt very lucky to be able to access the centre. It was viewed as a unique offering and there was a sense of great privilege that it was available in their locality. Beyond the exercise facilities, there was appreciation for the wider services available including assistance with finances, relaxation sessions for carers, high-quality catering and professional entertainers such as singers and comedians. The combined package facilitated motivation and enabled people to explore their boundaries in a safe environment;

*I think a lot of it is partly the socialisation but also the machines because he’s really trying hard with them, aren’t you? And again, like walking about and it’s easy to say at home “do your exercises” but it’s motivating yourself to do it, whereas here, you come along and have to. (Colin‘s partner)*

The opportunity to socialize with other people who have shared the experience of stroke is important;

*There’s the talking to other people; just talking to other people who have similar symptoms to me. (George, 62)*

Mike saw the centre as being pivotal in his life following stroke. His partner went out to work and he felt that he would be bereft if the centre was not available;

*I’ve come out of my shell more when I’m here … .without it? It would have been a big heartache … .stuck at home, stuck in the house. (Mike, 53)*

Other participants shared the sentiment that it would be difficult to fill time if the centre were not available. George recalled instances when bad weather had prevented him from attending;

*And not coming here means there’s a day to do nothing, I look to try and do bits of things to do at home. (George, 62).*

Wendy described how her second stroke had occurred whilst she was at the centre. She attributes her survival of this to the quick and professional action taken by the site team who ensured rapid hospital admission where she received thrombolysis.

### A renewed identity

Both female participants had taken on supporting roles at the centre as volunteers which generated a sense of renewed identity. Wendy explained that she felt very proud to be associated with the centre and its members;

*It’s strange because you feel like you’re living a completely different life, and I know this is going to sound really, really strange, but I feel more contented now because I’m not striving to achieve this, striving to achieve that.(F2)*

A complete faith in the people who founded and managed the centre was described and Liz felt delighted to be able to “give something back.” She reflected on the impact that the centre and equipment had on people’s lives and the positive changes observed amongst members, specifically mentioning one individual;

*He came first in a wheelchair, and now he walks up from the car park, with his stick … … because he’s been pushed on by what he can actually achieve and didn’t know he could. (Liz, 42)*

It was evident that membership at the centre represented an important component of overall identity for all participants. It had triggered the evolution of a renewed persona and enabled people to view stroke from a different perspective. Alongside the evolution of a “new self”, some participants expressed recovery of their “old self.” The loss of autonomy associated with early stroke had been restored;

*It’s good to feel more like me again (Mike, 53)*

The symbiotic relationship between members and the centre was summarized in a closing statement;

*You’re the one it’s happening to and if you can’t motivate yourself, I mean they do it here for you, they give you all the motivation you need. But you have to take it away with you as well. (Wendy, 68).*

In summary, the centre symbolized a turning point for all eight participants. They described a transition from the vulnerability associated with early stroke to an emergence of a renewed self. The benefits attributed to using the exercise equipment were viewed in the context of the centre as a holistic offering. The perceived social and physical changes were interwoven, illustrating the importance of the environment and setting when evaluating exercise interventions for PwS. There was some indication that mobility influenced the perceived physical effects of using PAE equipment; people with limited mobility reported strength gains whilst independently mobile participants associated the equipment with improved movement patterns. Female participants emphasized changes in participation and life roles associated with membership at the centre; in contrast, some male participants had experienced frustration during early rehabilitation and were determined to continue a trajectory of physical improvement. Cross-case analysis did not identify any clear trends associated with age, time since stroke or previous occupation and the reported experience of using the PAE equipment. The accessibility of the equipment when compared to conventional gymnasiums was emphasized, advancement of the software to generate feedback on user effort was suggested.

## Discussion

This study explored the personal experiences and perceived effects of engagement with PAE at a community-based venue for PwS. The application of interpretative phenomenological methodology generated a deeper understanding of engagement with PAE amongst PwS and the complex inter-relationship between venue membership and exercise facilities. Application of the hermeneutic cycle through IPA facilitated exploration of PAE in the broader context of adjustment to life with stroke and the venue in which the equipment was sited.

The participants in this study vividly recalled stroke as a sudden and devastating event, following which they transitioned through a health system which comprised hospital-based care followed by community rehabilitation. Previous phenomenological stroke research indicated that PwS recall feeling safe but disempowered within the hospital environment (Garrett et al., 2011). The findings from this study suggest that hospital care engendered an external locus of control. Rehabilitation was perceived in extrinsic terms as an occasional intervention administered to the participants, rather than an integral component of the care provided.

The participants in this study described meaningful achievements in terms of improved mobility within the first few weeks of returning home following stroke; this corroborates with quantitative evidence which reported increased physical activity levels amongst PwS following discharge home from hospital (Kerr et al., 2016). A positive team dynamic between the participants and rehabilitation team evolved and rehabilitation goals were focussed on the home environment and family. The participants in this study recalled improved confidence and sense of autonomy back in their home environment, which may reflect a process of reconnection as previously described by Pringle et al. (2013). However, uptake of exercise was not directly considered at this point in the recovery pathway.

Termination of health service rehabilitation was associated with feelings of abandonment and isolation. The findings of this study indicate a distinct need in the UK to ensure that PwS are further supported in accepting the termination of health service rehabilitation and access guidance in the uptake of community-based, third-sector services.

Challenges associated with timely and effective provision of information to PwS have been widely documented (Clague-Baker et al., 2017; O’Connell et al., 2009). The findings of this study indicate that the participants could not recall receiving advice on lifestyle, exercise or continued support services, and were in congruence with previous qualitative findings which found that people felt that there was nowhere else to go (Schouten et al., 2011). Miller et al. (2019) recognized current shortfalls in supporting PwS transition between different phases of the recovery pathway and outline five recommendations which include appointment of transition specialists and partnerships with community wellness programmes.

Several participants in this study recalled conversations with healthcare professionals pertaining to recovery plateau and this invoked feelings of anger and frustration. Participants in this study described ongoing physical gains several years after the onset of stroke and anticipated an indefinite trajectory of recovery. Exercise interventions are associated with improvements in motor performance, function and participation amongst people with chronic stroke (Linder et al., 2019). The concept of a recovery plateau following stroke has been repeatedly challenged (Page et al., 2004; Sun et al., 2015), but the findings of this study suggest that the term is still used in some areas of clinical practice. Dispelling the myth of recovery plateau amongst clinicians, general public end users, and media might empower and motivate PwS to engage in long-term exercise rehabilitation (Sun et al., 2015).

The uptake of PAE was synonymous with initiating membership at the stroke centre for nearly all of the participants. The exercise equipment represented a key incentive for joining the centre, although the value of the social dimension was emphasized by all participants. Recommendations for advancing the PAE equipment focussed on the development of effort detection software to enable users to gain feedback regarding their physical performance on an upgraded version of the console. The PAE mechanism enabled people with varying physical ability to participate on an equal footing which facilitated positive comparison and reciprocal support. Previous research has reported that long-term engagement with exercise following stroke required internally and externally sourced motivation (Scorrano et al., 2018), the expectations of other people enhanced commitment to exercise (Signal et al., 2016) and exercise participation in a venue was more motivating than the home environment (Poltawski et al., 2015). In-keeping with these previous findings, the value of positive peer support in a motivating and supportive setting was emphasized in the current study.

Several participants commented that PAE had made them feel fitter, stronger and more confident, with one participant commenting that she had restored her identity as “fun grandma.” Muscle weakness and aerobic deconditioning are a known consequence of stroke (Galloway et al., 2019; Ryan et al., 2017; Scherbakov et al., 2013). Combined aerobic and resistance training programmes are effective at improving physical function for PwS (Marzolini et al., 2018). However, the ability to generate and sustain repeated or resisted movements on the paretic side is a prerequisite for participation in conventional exercise interventions. Treatment strategies devised to address this clinical challenge include Functional Electrical Stimulation (FES) combined with cycling (Shariat et al., 2019) and robotic exoskeleton training (Stoller et al., 2013).

The combined reciprocal, bilateral movements which occur during PAE were likely to stimulate a physiological response in the aerobic, sensory and muscular systems; this might have contributed towards the perceived improvements in mobility and function reported in this study. The accessibility of the PAE machines was also identified as a key attribute of the equipment. Five of the eight participants were of working age but stroke-related impairments had enforced early retirement, and 75% of the participants in this study had a FAC score of three or less. Those participants with low FAC scores identified improved muscle strength as a perceived effect of using the equipment. The PAE machines were considered preferable to conventional gym equipment due to the variety and perceived safety. The need for inclusive and accessible exercise interventions for people with very limited mobility following stroke has been highlighted (Saunders et al., 2016).

Inclusion of non-ambulant PwS in exercise trials has been limited and mostly centred on assisted walking interventions (Lloyd et al., 2018). PAE might represent a feasible option for the inclusion of people with severe motor impairment in future stroke research.

An awareness of neuroplasticity and improved motor control was applied to the experience of PAE by some participants with higher FAC scores; perceived changes in their ability to control movement were associated with PAE. One participant (M3) described how his preferred machine changed according to his kinesiological goals. Assisted pedalling devices are available in many rehabilitation settings and some selected leisure venues (Kerr et al., 2019). The seated PAE equipment explored in this study may stimulate recovery of sagittal, frontal and transverse motor responses through the range of bimanual and bilateral movements assisted by the equipment. However, the space implications associated with accommodating the range of equipment may preclude smaller venues. Future research should determine whether seated PAE machines do enhance outcome in comparison to assisted pedalling devices and examine which machines may add most benefit.

The functional gains associated with PAE in this study might be at least partially attributable to neuroplastic adaptation triggered through engagement with assisted exercise. Assisted cycling enhanced neuroplastic potential and motor coordination for PwS (Holzapfel et al., 2019). Assisted exercise prior to physical rehabilitation interventions has been associated with enhanced neuroplastic potential (Linder et al., 2019). Some of the participants in this study also accessed the physiotherapy service available at the centre; service development could focus on implementing PAE to prepare people for therapeutic interventions.

Perspectives on optimal speed settings when using PAE equipment were varied amongst the participants as some believed that a fast pace generated a more intensive workout whilst others perceived the slow settings to be harder. Assisted cycling intervention protocols have stipulated a faster pace of movement than would be voluntarily initiated by the participant (Holzapfel et al., 2019; Linder et al., 2019) and this was deemed to enhance neuroplasticity and improve motor coordination. However, it may be hypothesized that improvements in muscular endurance and postural control could be more effectively stimulated through slowly paced assisted movement. Quantitative investigation is required to explore the physiological responses to PAE correlated with adjustment to speed settings.

The psychosocial benefits of PAE at the stroke centre were expressed by all participants, some of whom described a return of their pre-stroke sense of self, whilst others embraced a new positive identity following stroke. Although two participants had taken on voluntary roles at the venue; of the five participants who were of working age, none had felt able to resume their pre-stroke employment. Previous investigation into the experience of stroke amongst younger persons explored the devastating sequalae associated with loss of work in terms of self-identity, financial security and family role (Kuluski et al., 2014). The eclectic range of support services offered at the stroke centre precluded attributing psychosocial changes to PAE in isolation; the value of a combined package in an inclusive environment was clearly emphasized. Venue-based conventional exercise interventions following stroke were associated with restoration of confidence and self-efficacy, precipitating resumption of valued activities and recovery of vocational or family roles (Young et al., 2019). The participants in this study shared this perspective, indicating that type of exercise is not a strong determinant of psychosocial response.

### Methodological quality

The role of the researcher in IPA is to invite the participant to share their sense-making of life experiences, and in turn, through linguistic and conceptual interpretation, it is the task of the research team to make sense of the data generated (Smith, 2018a). To this end, our study was consistent with IPA; a strength of this paper was the exploration of pre-stroke lifestyle and experiences of early care to capture the shift from the pre-reflective sensory experience to reflective awareness of illness (Smith, 2018a). This depth of context highlighted the multi-faceted nature of adjustment to life with stroke and the complex interface, which exists between intrinsic and extrinsic drivers.

The study sample comprised participants from one specialist venue, which represented a unique facility for the local stroke population and the focus on PAE represents a novel contribution. Convergence in the findings facilitated recognition of common experiences and generated a foundation from which recommendations for service implementation and equipment advancement will be developed. The interpretation generated through the broader exploration of stroke onset and recovery corroborated with previous qualitative research, indicating attainment of theoretical generalizability between current findings and established perspectives (B. Smith, 2018b). However, all participants identified as being white British and there was no representation from low socioeconomic groups, which is possibly because of the membership fee required to access to the facilities. All participants described active lifestyles prior to the onset of stroke and had self-initiated their programme of PAE. Negative case analysis (Morse, 2015) may have captured an insight into the experiences of people who have disengaged from PAE and is identified as a future research priority in this field.

The lead author has a specific interest in PAE and the potential for “pink elephant” bias was recognized at inception of the study (Morse, 2015). The lead author kept a reflective journal and engaged all members of the research team in frequent debriefing sessions to facilitate reflexivity in the development of the research design, data analysis and synthesis of findings. The lead author sought to build a relationship with participants through repeated visits to the venue and a familiarization meeting prior to the scheduled interview. However, it is debatable as to whether this constituted a true prolonged engagement or persistent observation (Houghton et al., 2013). Repeated interviews or ethnographic observation of user engagement with PAE at the venue would have enriched the data collected.

### Future research actions and recommendations

The application of IPA during data analysis has been the catalyst to understanding the lived experience of PwS and explored transition from NHS rehabilitation to a third sector venue. PAE is a novel intervention for PwS and to the author’s knowledge this is the first in-depth qualitative exploration of the experience of using PAE in PwS. Perceived benefits were reported alongside suggestions for advancement of the machines including the development of effort detection technology to quantify user effort and enable operators, clinicians and researchers to record user performance. Suggestions for advancement of the machines have been realized through the securing of research funding with the remit to implement a participatory design and usability project to upgrade the software on PAE machines. This technology will enable more robust quantitative evaluation of the impact of PAE and enable differentiation between psychosocial influences and physical impact of the intervention.

The perspectives of people who opt out of exercise interventions following stroke continue to be an under represented population in the published evidence (Young et al., 2019). Future research should explore the perspectives of people who have discontinued a programme of PAE which will be a future area of enquiry for the research team and we encourage others to do the same.

## Conclusion

This study explored the lived experience and perceived effect of PAE amongst ambulant and non-ambulant PwS in a third sector stroke venue. It was anticipated that a deeper understanding of the lived experience of using the PAE equipment in the community would facilitate insight into outcomes of most importance to users of the equipment and guide future service and product development, which has been realized.

Participants recruited to the study recalled frustration on the termination of routine rehabilitation and sought a continued trajectory of physical recovery through engagement with PAE. The exercise equipment was associated with reported improvements in strength, movement and function but it was suggested that the equipment could be enhanced through the introduction of software designed to provide feedback on detected user effort. Use of the equipment was synonymous with the wider experience of membership at the venue, which was associated with regaining a renewed sense of identity following stroke.

## Data Availability

Raw data generated during this research study is stored in audio and electronic written format at Sheffield Hallam University. Derived data supporting the findings of this study are available from the corresponding author (RY) on request.

## Geolocation

North England, UK

## Appendix 1. Topic Guide

**Table:**

· **Can you tell me about your stroke?** o **When did it happen?** o **What do you remember about your early recovery?** · **Before your stroke how active were you? What activities did you enjoy?** · **How did your stroke affect the exercise or activities that you could do?** · **When did you start coming to the centre?** **What facilities do you use here?** · **What do you want to achieve through exercise?** · **What are your thoughts on exercising with power assisted equipment?** · **Do you think the machine assisted exercise has had any effect on your life outside the centre, for example**: · **Hobbies** · **Vocational activities** · **Family roles** · **Social life** · **Have you got any ideas or suggestions to enhance or develop the exercise equipment?** · **Have you any ideas for developing or improving your exercise programme?** · **What are your future exercise goals and plans?**
